# Adenosine reduces sinoatrial node cell action potential firing rate by uncoupling its membrane and calcium clocks

**DOI:** 10.3389/fphys.2022.977807

**Published:** 2022-11-24

**Authors:** Ashley N. Wirth, Kenta Tsutsui, Victor A. Maltsev, Edward G. Lakatta

**Affiliations:** Laboratory of Cardiovascular Science, Intramural Research Program, National Institute on Aging, NIH, Biomedical Research Center, Baltimore, MD, United States

**Keywords:** sinoatrial node (SAN), adenosine, coupled-clock pacemaker system, calcium release, sarcoplasmic reticulum (SR), cardiac arrhythmia, sinus node arrest, sick sinus syndrome

## Abstract

The spontaneous action potential (AP) firing rate of sinoatrial nodal cells (SANC) is regulated by a system of intracellular Ca^2+^ and membrane ion current clocks driven by Ca^2+^-calmodulin-activated adenylyl cyclase-protein kinase-A signaling. The mean AP-cycle length (APCL) and APCL variability inform on the effectiveness of clock coupling. Endogenous ATP metabolite adenosine binds to adenosine receptors (A_1_, A_3_) that couple to G_i_ protein-coupled receptors, reducing spontaneous AP firing rate *via* G_βγ_ signaling that activates I_KAch,Ado_. Adenosine also inhibits adenylyl cyclase activity *via* G_αi_ signaling, impacting cAMP-mediated protein kinase-A-dependent protein phosphorylation. We hypothesize that in addition to I_KAch,Ado_ activation, adenosine impacts also Ca^2+^ *via* G_αi_ signaling and that both effects reduce AP firing rate by reducing the effectiveness of the Ca^2+^ and membrane clock coupling. To this end, we measured Ca^2+^ and membrane potential characteristics in enzymatically isolated single rabbit SANC. 10 µM adenosine substantially increased both the mean APCL (on average by 43%, *n* = 10) and AP beat-to-beat variability from 5.1 ± 1.7% to 7.2 ± 2.0% (*n* = 10) measured *via* membrane potential and 5.0 ± 2.2% to 10.6 ± 5.9% (*n* = 40) measured *via* Ca^2+^ (assessed as the coefficient of variability = SD/mean). These effects were mediated by hyperpolarization of the maximum diastolic membrane potential (membrane clock effect) and suppression of diastolic local Ca^2+^releases (LCRs) (Ca^2+^-clock effect): as LCR size distributions shifted to smaller values, the time of LCR occurrence during diastolic depolarization (LCR period) became prolonged, and the ensemble LCR signal became reduced. The tight linear relationship of coupling between LCR period to the APCL in the presence of adenosine “drifted” upward and leftward, i.e. for a given LCR period, APCL was prolonged, becoming non-linear indicating clock uncoupling. An extreme case of uncoupling occurred at higher adenosine concentrations (>100 µM): small stochastic LCRs failed to self-organize and synchronize to the membrane clock, thus creating a failed attempt to generate an AP resulting in arrhythmia and cessation of AP firing. Thus, the effects of adenosine to activate G_βγ_ and I_KACh,Ado_ and to activate G_αi_, suppressing adenylyl cyclase activity, both contribute to the adenosine-induced increase in the mean APCL and APCL variability by reducing the fidelity of clock coupling and AP firing rate.

## 1 Introduction

Cardiac sinoatrial nodal (SAN) pacemaker cells (SANC) generate spontaneous action potentials (APs) that initiate each heartbeat. Adenosine (ado) is an endogenous cardiac metabolite, generated *via* the enzymatic hydrolysis of AMP or S-adenosyl homocysteine (Schrader, 1983; Shen and Kurachi, 1995). Ado concentration increases when ATP becomes reduced during metabolic stress and works to slow heart rate, thereby reducing energy consumption to protect the heart (West and Belardinelli, 1985; Belardinelli et al., 1988).

Ado has been previously shown to slow SAN impulses and delay SAN conduction (Drury and Szent-Gyorgyi, 1929). Applications of ado to single isolated SANC reduce the AP firing rate (Belardinelli et al., 1988; Headrick et al., 2011) *via* ado-activated A_1_ and A_3_ receptors signaling that like muscarinic receptor M_2_ stimulation leads to activation of inhibitory GTP-binding protein to dissociate into its G_αi_ and G_βγ_ subunits. These effects, respectively, result in both the inhibition of adenyl cyclase (AC) and activation of GIRK4 K^+^ channels that conduct I_KACh_ and IKAdo currents (I_KAch,Ado_) that hyperpolarize the SANC membrane potential (Kurachi et al., 1986; Logothetis et al., 1987; Belardinelli et al., 1988; Shen and Kurachi, 1995; Dessauer et al., 1996; Jurevicius and Fischmeister, 1996; Cabrera-Vera et al., 2003; Okumura et al., 2003; Lyashkov et al., 2009). Ado is also known as an anti-adrenergic agent [review (Belardinelli et al., 1995)], and at a concentration of 50 μM it attenuates the increases in both I_CaL_ and I_f_ caused by β-adrenergic receptor stimulation (Belardinelli et al., 1988). However, ado in sub-micromolar concentrations inhibits I_f_ in the absence of β-adrenergic stimulation and slows pacemaking in SANC similar to acetylcholine (ACh) (Zaza et al., 1996b).

It was shown that when both I_KAch,Ado_ and/or I_f_ are blocked (tertiapin-Q and/or Cs), the AP firing rate reduction in rabbit SANC still remains substantial within the broad range of physiological concentrations of ACh [Figure 3 in (Lyashkov et al., 2009)]. Furthermore, in the absence of β-adrenergic receptor stimulation, ACh in physiological concentration of 0.1 μM has almost no effect on I_CaL_ amplitude [<2%, (Zaza et al., 1996a)]. Thus, previous experimental studies of ACh responses have indicated that membrane currents (I_KACh,Ado_, I_f_, and I_CaL_) are not the only components of the complex effect ACh (and hence Ado) on AP firing rate.

The concept that SANC AP firing is regulated by a coupled-oscillator system, driven by Ca^2+^-calmodulin activated AC-protein kinase-A (PKA) and CaMKII signaling is widely accepted (Lakatta et al., 2010; Weiss and Qu, 2020). The sarcoplasmic reticulum (SR) operates as an intracellular Ca^2+^ oscillator or “Ca^2+^ clock”, and it couples to an ensemble of voltage and time-dependent surface membrane current oscillators “Membrane clock”. The Ca^2+^ clock generates spontaneous, rhythmic diastolic local Ca^2+^ releases (LCRs), that activate inward Na^+^/Ca^2+^ exchanger current (I_NCX_) (Bogdanov et al., 2001; Vinogradova et al., 2004), which partners with I_f_ and I_CaT_ to initiate the diastolic depolarization (Lyashkov et al., 2018). Feed-forward signaling involving low-voltage activated Ca^2+^ channels (Ca_v_1.3) (Torrente et al., 2016), Ca^2+^-induced Ca^2+^ release (CICR), and continued spontaneous LCRs from the SR form an ensemble Ca^2+^ signal that accelerates diastolic membrane depolarization *via* I_NCX_ activation (Lyashkov et al., 2018). When the membrane potential (V_m_) depolarizes sufficient to open Ca_v_1.2 L-type Ca^2+^ channels, an AP is ignited, which (*via* CICR) induces a relatively synchronous activation of Ca^2+^ release channels (ryanodine receptors), resulting in a global cytosolic Ca^2+^ transient (Fabiato, 1983; Stern et al., 1999). AP firing itself, *via* its effects to regulate intracellular Ca^2+^, the “oscillatory substrate” of the Ca^2+^ clock (Maltsev et al., 2006), affects Ca^2+^-ligand function of proteins of both clocks and thus affects clock coupling (Maltsev and Lakatta, 2009). The average AP firing rate and AP cycle length (APCL) inform on the fidelity of clock coupling: when clock coupling decreases, the mean AP firing rate is reduced and the variability of APCL increases (Yaniv et al., 2014b; Moen et al., 2019).

Within the coupled clock paradigm, the effect of ACh to slow down the SANC rate has been systematically evaluated both experientially and in numerical model simulations (Lyashkov et al., 2009; Maltsev and Lakatta, 2010). In addition to the aforementioned effect to activate GIRK4 channels, leading to membrane hyperpolarization (DiFrancesco and Tromba, 1988; Demir et al., 1999), cholinergic receptor stimulation also inhibits cAMP-PKA signaling that has a direct effect to reduce intracellular Ca^2+^ cycling, reducing Ca-calmodulin-activated AC-PKA-CAMKII signaling (Lyashkov et al., 2009). This effect, in conjunction with the activation of GIRK4 K^+^ channels, reduces the mean AP firing rate (Lyashkov et al., 2009) and increases AP beat-beat variability (Yaniv et al., 2014a). Reduced AP firing reduces net Ca^2+^ influx and therefore intracellular Ca^2+^, the “oscillatory substrate” of the Ca^2+^ clock. Thus, ACh reduces functions of the clocks and the effectiveness of the clocks coupling *via* several intertwined Ca^2+^ and voltage-dependent mechanisms.

With respect to ado, while the membrane clock aspects of its action have been thoroughly studied for many decades, its impact on Ca^2+^ signaling in terms of coupled clock model is expected but has not been evaluated and remains the missing piece in our knowledge of the complex effect of ado to slow down the pacemaker system. On the other hand, although ado and ACh act *via* different receptors (A_1_/A_3_ and M_2_, accordingly), they both exert their downstream effects *via* a signaling cascade that involves the same G_i_ protein coupling (Lyashkov et al., 2009) that inhibits AC activity *via* a G_αi_ effect and activates GIRK4 K^+^ channels *via* G_βγ_ effect. Thus, the present study tested the hypothesis, that ado, like ACh, effects, in addition to membrane hyperpolarization, also changes in LCRs that contribute to the bradycardic effect of ado by reducing coupling of the clocks.

## 2 Material and methods

### 2.1 Single cell preparation

SANC were isolated from male rabbits in accordance with NIH guidelines for the care and use of animals, protocol # 34-LCS-2019 (as previously described) (Vinogradova et al., 2000). New Zealand White rabbits (Charles River Laboratories, United States) weighing 2.8–3.2 Kg were anesthetized with sodium pentobarbital (50–90 mg/kg). The heart was removed quickly and placed in solution containing (in mM): 130 NaCl, 24 NaHCO_3_, 1.2 NaH_2_PO_4_, 1.0 MgCl_2_, 1.8 CaCl_2_, 4.0 KCl, 5.6 glucose equilibrated with 95% O_2_/5% CO_2_ (pH 7.4 at 35°C). The SAN region was cut into small strips (∼1.0 mm wide) perpendicular to the crista terminalis and excised as reported previously (Vinogradova et al., 2000). The final SA node preparation, which consisted of SA node strips attached to the small portion of crista terminalis, was washed twice in nominally Ca^2+^-free solution containing (in mM): 140 NaCl, 5.4 KCl, 0.5 MgCl2, 0.33 NaH_2_PO_4_, 5 HEPES, 5.5 glucose, (pH = 6.9) and incubated on shaker at 35°C for 30 min in the same solution with the addition of elastase type IV (0.6 mg/ml; Sigma, Chemical Co.,) collagenase type 2 (0.8 mg/ml; Worthington, NJ, United States), Protease XIV (0.12 mg/ml; Sigma, Chemical Co.,) and 0.1% bovine serum albumin (Sigma, Chemical Co.,). The SA node preparation was next placed in modified Kraftbruhe (KB) solution, containing (in mM): 70 potassium glutamate, 30 KCl, 10 KH_2_PO_4_, 1 MgCl_2_, 20 taurine, 10 glucose, 0.3 EGTA, and 10 HEPES (titrated to pH 7.4 with KOH), and kept at 4°C for 1 h in KB solution containing 50 mg/ml polyvinylpyrrolidone (PVP, Sigma, Chemical Co.,). Finally, cells were dispersed from the SA node preparation by gentle pipetting in the KB solution and stored at 4°C.

### 2.2 High speed 2D Ca^2+^ signal imaging

Ca^2+^ dynamics within isolated single rabbit SANC were measured by 2D imaging of the fluorescent Ca^2+^ indicator, Fluo-4. Cells were loaded with 5 μM Fluo-4AM (Thermo Fisher, United States) for 15 min at room temperature. Fluo-4AM was subsequently washed out of the chamber with bathing solution contained the following (in mM): NaCl 140, HEPES 5, NaH_2_PO_4_ 0.33, KCl 5.4, MgCl_2_ 1.0, glucose 5.5, CaCl_2_ 1.8; titrated to pH 7.35 with NaOH. Ca^2+^ signals were measured within the ensuing 30 min at 35°C ± 0.1°C. Temperature was controlled by an Analog TC2BIP 2/3Ch bipolar temperature controller from CellMicroControls (United States), which heated both the glass bottom of the perfusion chamber and the solution entering the chamber (*via* a pre-heater). It is important to note that Ca^2+^ imaging was not performed immediately after cells were taken from 4°C KB solution, but following an adaptation time of 20–30 min, during which cells settled in the perfusion chamber, attach to the glass, and were loaded with the Ca^2+^ indicator. Ca^2+^ signals were recorded only from rhythmically firing SANC at baseline (CV<10%). Thus, the measured cells were truly pacemaker cells by their classical morphological shapes and by the presence of rhythmic spontaneous beating, and therefore other cell types (fibroblasts, neurons, etc.,) were excluded by these criteria.

Fluo-4 fluorescence was detected using a high-speed PCO. edge 4.2 CMOS camera (100 frames-second, with an 13.2 mm square sensor of 2048 × 2048 pixels resolution) mounted on a Zeiss inverted microscope (Carl Zeiss, Inc. Germany) with a x63 oil immersion lens and a fluorescence excitation light source (CoolLED pE-300-W, BioVision Technologies, Inc. PA, United States). Fluo-4 fluorescence excitation (blue light, 470/40 nm) and emission light collection (green light, 525/50 nm) were performed using the Zeiss filter set 38 HE. To avoid phototoxicity, Fluo-4 was excited only for short periods of time (<20 s) (Monfredi et al., 2013; Kim et al., 2018). Data acquisition was performed using PCO camware 64 (PCO AG, Germany).

### 2.3 Membrane potential recording

V_m_ was measured in the current clamp configuration using an Axopatch 200B amplifier (Molecular Devices). Patch pipette resistances ranged between 3–5 MΩ, and pipettes were filled with a solution containing (in mM): K^+^ gluconate 120, NaCl 5, MgATP 5, HEPES 5, KCl 20; titrated to pH 7.2 with KOH. Amphotericin B (320 μM, Sigma-Aldrich A-4888) was added into the pipette solution as the pore-forming agent. The liquid junction potential was calculated by pClamp software (Molecular Devices) to be 13 mV and included in data analysis.

### 2.4 Computational analysis of LCRs and APs

**FIGURE 1:**
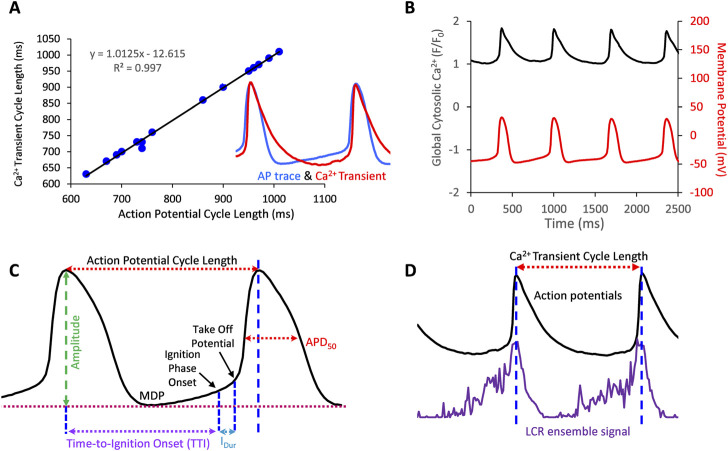
**(A)** Action potentials and AP-Induced Ca^2+^ transients are highly correlated in SANC in which both Ca^2+^ and V_m_ were simultaneously measured (17 cycles measured simultaneously). **(B)**, example of a simultaneous V_m_ and Ca^2+^ recording. **(C,D)** Illustrate V_m_ and Ca^2+^ parameters measured in this study.

We used an in-house custom program (“XYT Event Detector”) to objectively, automatically, and rapidly analyze the individual and ensemble behavior of the LCRs (Maltsev et al., 2017). The program yields detailed information about the number, timing, and size of individual LCRs. Specifically, The LCR timing is assessed as “LCR period” which is the time interval between the peak of the prior AP-induced Ca^2+^ transient peak and the onset of the LCR occurrence. The “LCR size” is given as the LCR full propagation path in µm^2^. The computer program also provides the LCR ensemble signal (i.e., the summation of all LCR Ca^2+^ signal areas occurring within a given time, Figure 1D). APs were analyzed by using another in-house custom program “AP analysis” (Lyashkov et al., 2007). In addition to traditional parameters APCL, APD50, MDP, and Take-Off potential, the program also provided AP ignition parameters introduced in (Lyashkov et al., 2018): Ignition duration (Idur), and Time-To-Ignition onset (TTI) (Figure 1C).

### 2.5 Experimental protocol

In some cells we measured APs only, and in others we measured Ca^2+^ signal only. V_m_ and Ca^2+^ were also recorded simultaneously in a subset of SANC. Both V_m_ and Ca^2+^ recordings were electronically synchronized. The V_m_ measurements and 2D Ca^2+^ signals were obtained before, during, and following washout of 10 µM ado (Sigma Aldrich, United States). We measured SANC APCL using two parameters: AP intervals or AP-induced Ca^2+^ transient intervals that were closely correlated (*R* ^2^ = 0.99, Figures 1A,B). The rhythmicity of SANC firing was assessed as the coefficient of variation (CV) of AP or Ca^2+^ transient intervals in time series of AP’s or AP-induced Ca^2+^ transient cycles. The mean LCR period was the averaged LCR period of 3-7 cycles at baseline and with ado.

### 2.6 Numerical modeling

To get insights into the complex effects of ado in the coupled-clock system, we provide results of numerical modeling described previously for ACh effects (Maltsev and Lakatta, 2010).

### 2.7 Statistics

Values are expressed as mean ± standard error. Ca^2+^ and electrophysiological measurements in control were compared to those in the presence of ado by one-way ANOVA, paired *t*-test or *via* Student’s *t*-test, as indicated in the Figure legends. *p*-value < 0.05 was considered statistically significant.

## 3 Results

### 3.1 AP firing rate and rhythm

**FIGURE 2:**
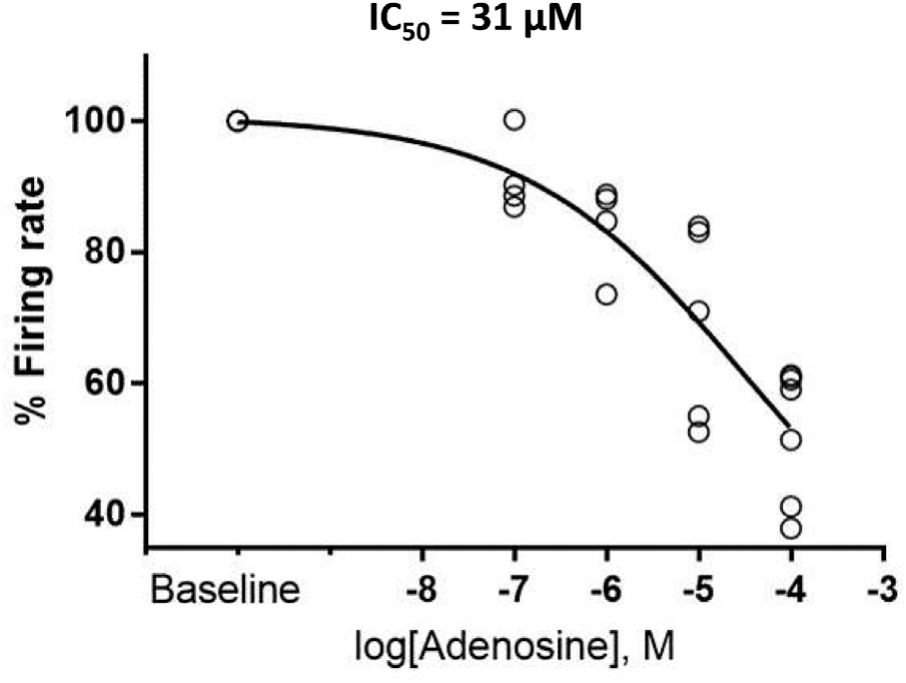
Adenosine slows the firing rate of rhythmically firing SANC in a dose-response manner. The IC_50_ for our dose response curve was 31 µM adenosine. (*n* = 4 for 100 nm and 1 µM adenosine, *n* = 5 for 10 µM adenosine, and *n* = 7 for 100 µM adenosine). The data of firing rate response were normalized to be between 0% and 100% of baseline and fitted to Variable slope model using GraphPad program https://www.graphpad.com/guides/prism/latest/curve-fitting/reg_dr_inhibit_normalized_variable.htm. The equation for this fitting function was: Y = 100/(1 + 10^((LogIC50-X)*HillSlope)].

**FIGURE 3:**
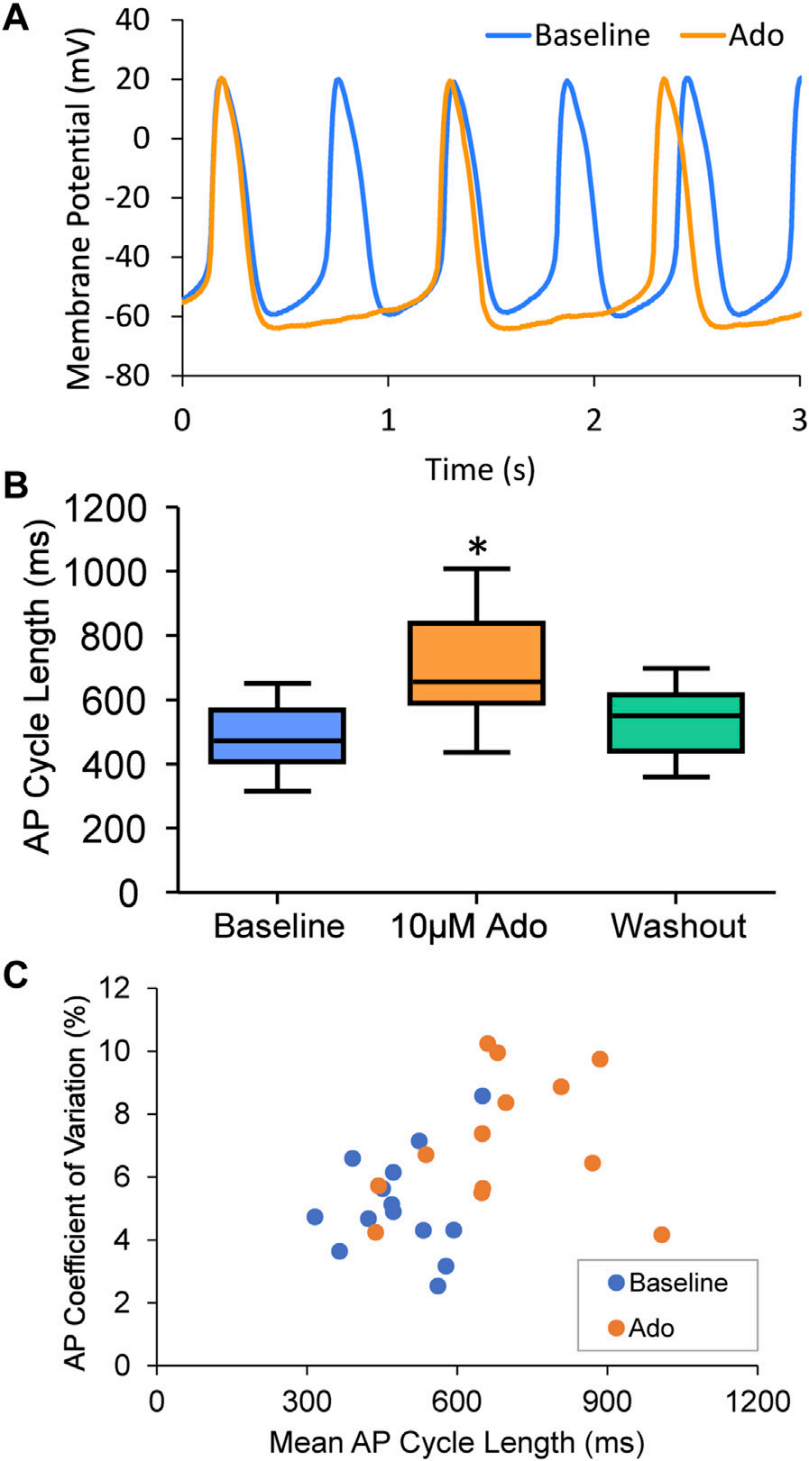
**(A)** an example of a cell that increased APCL from 577 ms at baseline to 1009 ms with adenosine and recovered to 597 ms with washout (washout not shown). **(B)** Statistical analysis of adenosine effect: SANC action potential cycle length (APCL) increased with adenosine and recovered with washout in SANC (*n* = 14). **(C)** APCL and coefficient of variation (CV) increased with adenosine. *p < 0.05 *via* one-way repeated measures ANOVA.

**FIGURE 4:**
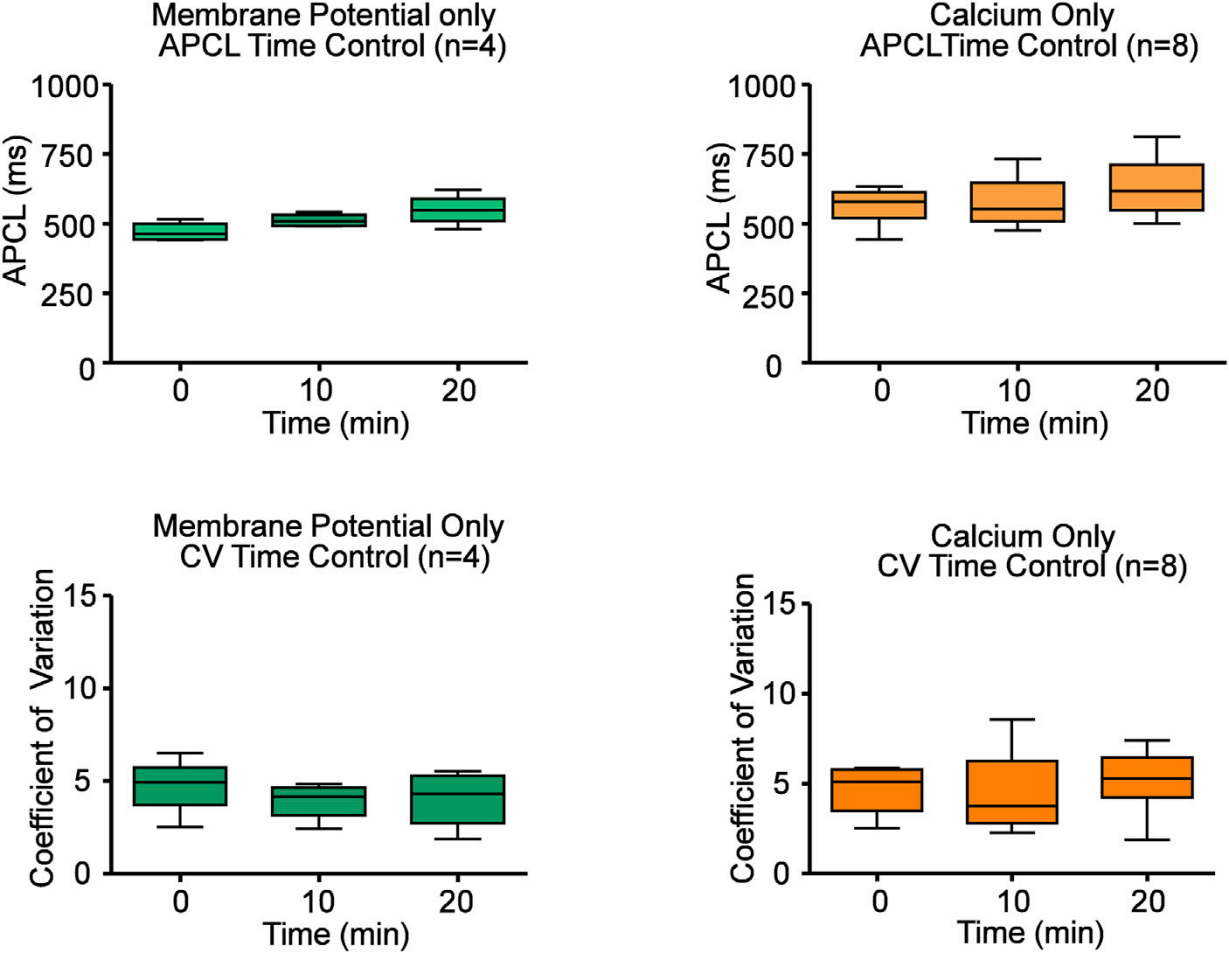
Time controls of Ca^2+^ and electrophysiology measurements show that SANC action potential cycle length (APCL) and coefficient of variation (CV) do not change with time. Repeated measures ANOVAs of Ca^2+^ and V_m_ measurements showed there was no significant time effect on SANC firing rate and CV within twenty minutes. Based on this data, all experiments were performed within twenty minutes.

Ado dose-dependently increased AP-induced Ca^2+^ transient cycle length (Figure 2; Table 1). Ado, at a concentration near its IC_50_ (10 µM) (Lou et al., 2014), increased APCL from 492 ± 88 to 687 ± 178 (Table 2). In response to 10 µM ado, all rhythmically firing SANC increased APCL, measured *via* V_m_ or Ca^2+^ signal, and most recovered with washout (Figure 3). The CV of AP also increased in response to 10 µM ado from 5.1% ± 1.7% to 7.2% ± 2.0% (Table 2). To confirm that the increase in APCL was due to ado effects, time controls of Ca^2+^ and V_m_ measurements were also conducted. There was no significant time effect on either rate of AP firing or AP-induced Ca^2+^ transients’ measurement within thirty minutes (Figure 4).

**TABLE 1:** Adenosine slows SANC firing rate in a dose-dependent manner. Calcium signal measurements of rhythmically firing SANC were taken before and after adenosine exposure. To minimize cell phototoxicity, different cells were used for each dose of adenosine. All SANC measured slowed down in response to adenosine. At each concentration of adenosine, the increase in APCL was significant (*p* < 0.05).

Parameters	Baseline (*n* = 20)	Adenosine 100 nm (*n* = 4)	Adenosine 1 µM (*n* = 4)	Adenosine 10 µM (*n* = 5)	Adenosine 100 µM (*n* = 7)
SANC APCL (ms)	408 ± 70	435 ± 43*	531 ± 65*	670 ± 277*	703 ± 222*
SANC APCL % Change	100	108 ± 7	120 ± 11*	150 ± 34*	184 ± 48*
Coefficient of Variation (%)	4.9 ± 2.9	7.2 ± 2.8	8.9 ± 6.7	12.1 ± 12.6	10.5 ± 7.9*

**TABLE 2:** Action potential parameters measured in this study. Action potential characteristics of SANC (Figure 1C) were measured before (Baseline), during, and after adenosine perfusion (Washout). The data obtained from 10 single SANC isolated from five rabbit hearts. *p < 0.05, compared to baseline by two-tailed paired *t* test. CL; cycle length max-to-max, CV; coefficient of variation, MDP; max diastolic potential, Ampl, amplitude; dVdt_max_, maximum upstroke velocity; TTI, time-to-ignition onset; IP, Ignition Potential; I_Dur_, Ignition Duration (from IP to TOP); Time to TOP (from MDP to TOP); TOP, take off potential.

Condition	*n*	CL (ms)	CV (%)	MDP (mV)	Ampl (mV)	dVdt_max_ (V/s)	APD_50_ (ms)	Mean DD slope (mV/s)	TTI (ms)	IP (mV)	I_Dur_ (ms)	TOP (mV)
Baseline	10	492 ± 88	5.1 ± 1.7	−64.1 ± 5.6	89.8 ± 9.1	3.7 ± 1.1	135 ± 28	43.0 ± 18	478.5 ± 164	−56.5 ± 8.1	24.6 ± 10.5	−59.0 ± 6.7
Ado	10	687 ± 178*	7.2 ± 2.0*	−67.7 ± 4.8*	97.2 ± 9.2*	3.9 ± 1.4*	141.6 ± 22.3	22.8 ± 9.8*	773.9 ± 498*	−57.0 ± 7.4	28.1 ± 13.6*	−59.4 ± 5.9
Ado,% of baseline	10	143 ± 33*	142 ± 36*	105 ± 4.0*	110 ± 7.8*	117 ± 14*	106 ± 15	58 ± 25*	150 ± 38*	101 ± 3.4	112 ± 16	101 ± 3.4
Washout	10	554 ± 102	5.0 ± 1.2	−66.1 ± 3.9	95.2 ± 9.2*	3.9 ± 1.2*	138.6 ± 20.9	42.0 ± 29.1	518.7 ± 143	−56.5 ± 8.0	26.4 ± 8.7	−58.5 ± 6.9
Washout % of baseline	10	109 ± 13	104 ± 31	104 ± 6.9	108 ± 9.5*	115 ± 12*	107 ± 19	100 ± 38	111 ± 16	100 ± 11	109 ± 25	101 ± 11

### 3.2 Membrane potential

Ado prolonged the APCL (Figures 3A,B) confirming prior observations (Belardinelli et al., 1988; Ren et al., 2003). In SANC in which only V_m_ was measured, the ado-induced increase in APCL was washable after 10 min (Figure 3B; Table 2). Ado significantly hyperpolarized maximum diastolic potential (MDP) and significantly increased AP amplitude, maximum upstroke velocity (dVdt_max_), and decreased the mean diastolic depolarization slope (Figure 3A; Table 2). The significant hyperpolarization of MDP and increases in APCL and dVdt_max_ in response to ado are also consistent with previous studies in isolated SANC (Ren et al., 2003). Concurrently, with the prolongation of mean APCL, APCL variability increased (Figure 3C; Table 2). Time to Ignition Onset (TTI), defined as the time period from the AP peak to the ignition phase onset (Figure 1C), shown to predict the APCL (Lyashkov et al., 2018) was also markedly prolonged by ado (Table 1). The ignition process itself was also slowed down evidenced by significantly longer Idur (Table 1).

### 3.3 Ca^2+^ transients

**FIGURE 5:**
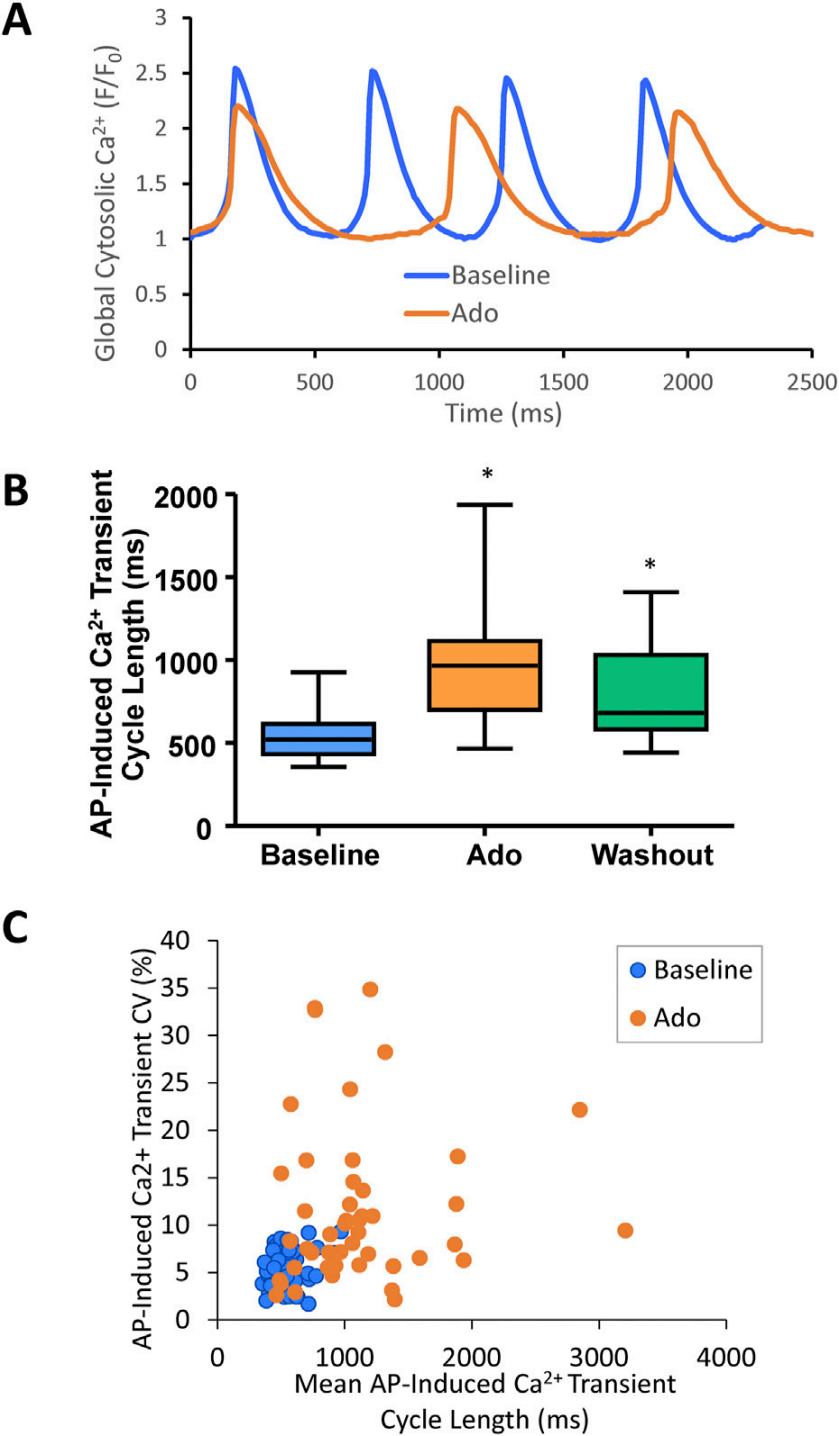
**(A)** an example of a cell that increased APCL from 480 ms at baseline to 888 ms with adenosine and recovered to 648 ms with adenosine and recovered to 648 ms with washout (washout not shown). **(B)** SANC AP-induced Ca^2+^ transient cycle length increased with adenosine and recovered partially with washout. (*n* = 46). **(C)** SANC AP-Induced Ca^2+^ transient cycle length and coefficient of variation (CV) increased with adenosine; *p < 0.05 *via* one-way repeated measures ANOVA.

An example of Ca^2+^ signal measurement in a representative cell is shown in Figure 5A. Ado increased the mean AP-induced Ca^2+^ transient cycle length and its cycle-to-cycle variability (Figures 5B,C; Table 3) to similar extents as for those measured *via* perforated patch clamp (Figure 3; Table 2)*.*

**TABLE 3:** Characteristics of AP-Induced Ca^2+^ transient and Local Ca^2+^ releases releases (LCRs) measured in this study. Ca^2+^ signal measurements in SANC before and during adenosine perfusion (*n* = 40). All measured cells were rhythmically firing at baseline. *p < 0.05, compared to baseline by two-tailed paired *t* test.

Condition	*n*	CL (ms)	CV (%)	Time to peak Ca^2+^(ms)	Peak Ca^2+^ (F)	Time to 90% decay (ms)	
Baseline	40	540 ± 129	5.0 ± 2.2	493 ± 115	367 ± 252	378 ± 114	
Adenosine	40	950 ± 331 *	10.6 ± 5.9 *	833 ± 338 *	301 ± 210 *	707 ± 335 *	
Adenosine % of Baseline	40	155 ± 56 *	200 ± 91 *	205 ± 137 *	84 ± 23 *	187 ± 72 *	

Condition	*n*	# Of LCR Events/Sec	LCR size (µM^2^)	LCR duration (ms)	Mean LCR ensemble (µM^2^)	Mean LCR period (ms)	LCR period SD (ms)
Baseline	40	146 ± 75	7.4 ± 2.6	16.6 ± 3.4	986 ± 437	370 ± 179	209 ± 107
Ado	40	157 ± 68	5.6 ± 2.2*	14.8 ± 2.6*	770 ± 358*	646 ± 320*	354.8 ± 171*
Ado % of Baseline	40	119 ± 42*	78 ± 19.0*	89.3 ± 6.5*	81 ± 22*	184 ± 83*	185 ± 88*

### 3.4 Effects of ado on spontaneous, local diastolic Ca^2+^ releases

**FIGURE 6:**
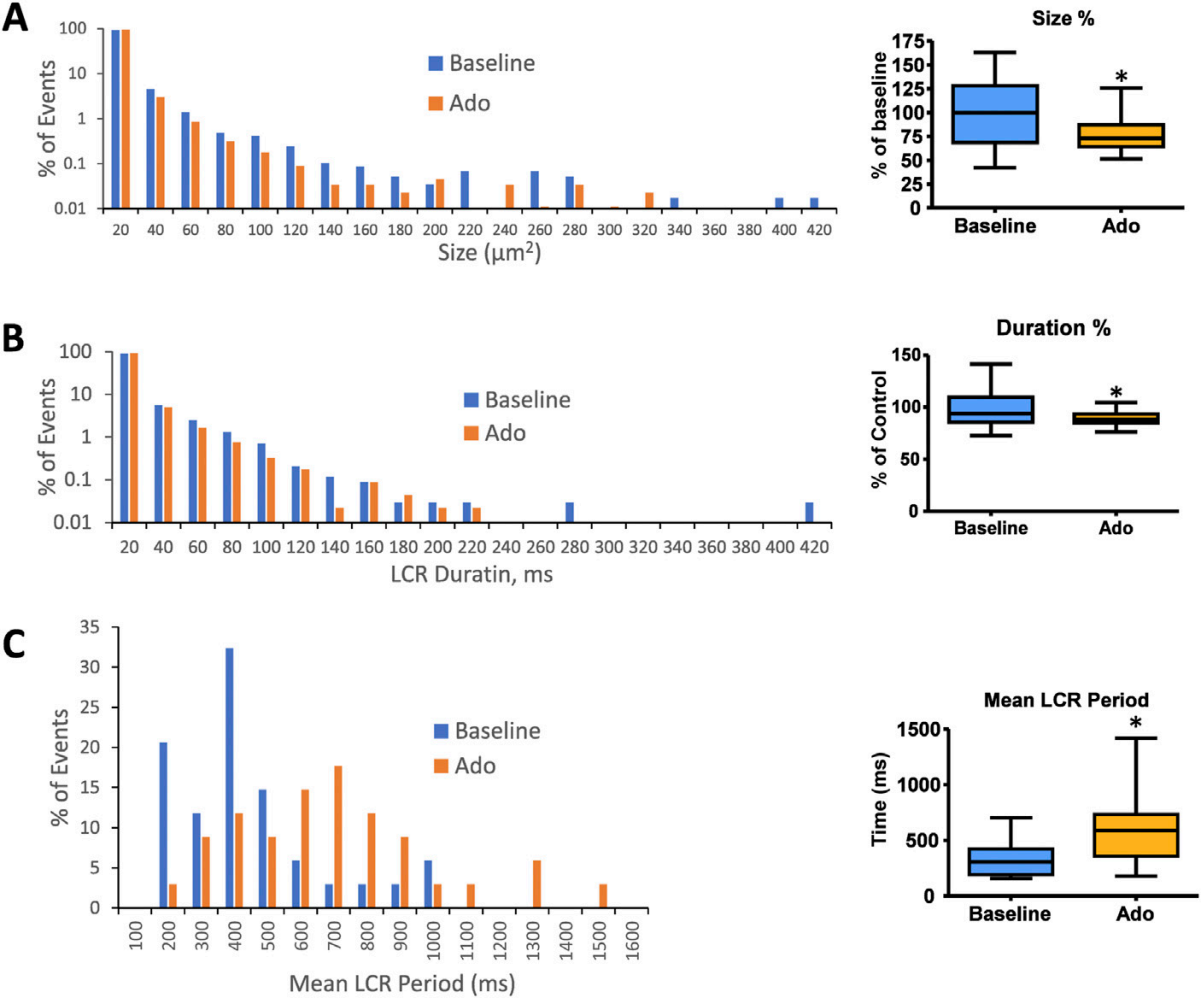
Histograms of LCR size **(A)**, LCR duration **(B)**, and mean LCR period **(C)** percentage distributions in all rhythmic SANC before and in the presence of adenosine (*n* = 34). Mean LCR period was the averaged LCR period of 3–7 cycles at baseline and with adenosine for each SANC. Mean LCR period also increased in response to adenosine; *p < 0.05 *via* one-way repeated measures ANOVA.

The results of statistical analysis of LCR characteristics are presented in Table 3 and illustrated in Figure 6. Ado did not change the mean number of diastolic LCR events (normalized for longer diastolic times) but reduced the mean LCR size, duration, and ensemble LCR Ca^2+^ signal. When expressed as a percent of control, ado also reduced the LCR size, duration, and ensemble Ca^2+^ signal but also increased the number of LCRs, consistent with the idea that LCRs becomes smaller and less synchronized (i.e., de-synchronized in space and time). With ado, there was an increase in the number and percentage of the smallest LCRs. Correspondingly, there was a decrease in larger LCRs. Ado also prolonged the mean LCR period, consistent with the idea that the period of the coupled-clock system increased.

It has been previously shown that a concurrent increase in beat-to-beat variability (CV) accompanied an increase in the mean AP-induced Ca^2+^ transient cycle length and mean LCR period in response to ivabradine, a funny current inhibitor (Yaniv et al., 2014b). The increase in mean AP-induced Ca^2+^ transient cycle length and mean LCR period in response to ado were also accompanied by the increased beat-to-beat variability (CV) of the LCR period and APCL (Figure 5C; Tables 3).

### 3.5 Clock uncoupling in response to ado

**FIGURE 7:**
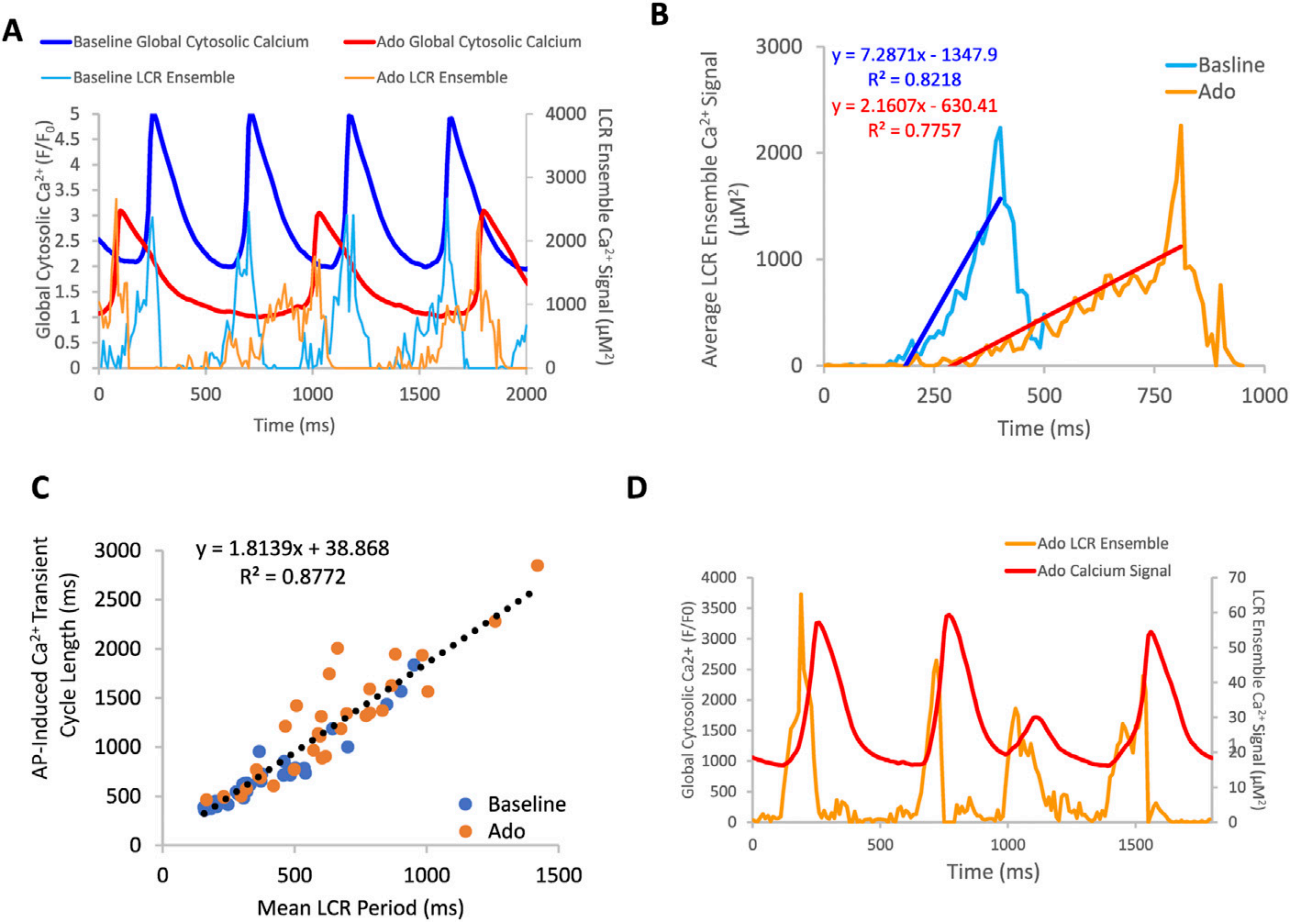
**(A)** an example of a rhythmically firing SANC that decreases in rate of LCR ensemble growth in response to adenosine. There is an overall decrease in global cytosolic Ca^2+^ with adenosine compared to baseline. Panel **(B)** depicts the average LCR ensemble growth rate of 5 cycles at baseline and with ado. The average LCR ensemble growth rate decreased to 42% of baseline with adenosine. This decrease reflects the changes in intracellular Ca^2+^ availability, manifesting in changes to LCR parameters. This results in an extended time period of Ca^2+^ cycling between AP-induced Ca^2+^ transients. Panel **(C)**, the correlation between mean LCR Period (3–7 beats) and AP-induced Ca^2+^ transient cycle length is reduced at longer cycle lengths. At baseline, there is a strong correlation between APCL and LCR period, indicating robust clock coupling (*n* = 35). Adenosine, in the same population of cells, the mean APCL and LCR period increased for many cells. For a subset of cells, mean APCL increased more than mean LCR period, indicating reduced fidelity of clock coupling indicated by the orange circles that digress up and leftward from the linear trendline (dashed line). The linear trendline includes baseline and ado values. Panel **(D)**, an example of a SANC with ado where LCR ensemble growth propagation was insufficient and failed to generate an AP, resulting in clock uncoupling and a “failed AP attempt”.

The effect of ado on LCR characteristics resulted in a reduction of the self-organized growth rate of diastolic LCR ensemble Ca^2+^ signal (Figure 7). At baseline, the majority of SANC showed a strong correlation between LCR period and AP-induced Ca^2+^ transient cycle length, indicating robust clock coupling in most cells (Figure 7C). In the presence of ado, the mean LCR period and Ca^2+^ transient cycle length increased from baseline but maintained the correlation between LCR period and AP-induced Ca^2+^ transient cycle length (Figure 7C). For a few cells that deviated from this correlation with ado, as the mean APCL increased in response to ado, a given LCR period was linked to a longer APCL than at baseline (Figure 7C), reflecting a reduction of the effectiveness of LCR signals to impact the timing of the next AP. This reduced effectiveness manifested as a “missed attempt” at synchronizing between the Ca^2+^ and membrane clock to generate an AP (Figure 7D). The cells that deviated rightward and upward the most from the correlation between LCR period and AP-induced Ca^2+^ transient cycle length with ado experienced the most uncoupling.

### 3.6 Simultaneous measurements of membrane potential and Ca^2+^

**FIGURE 8:**
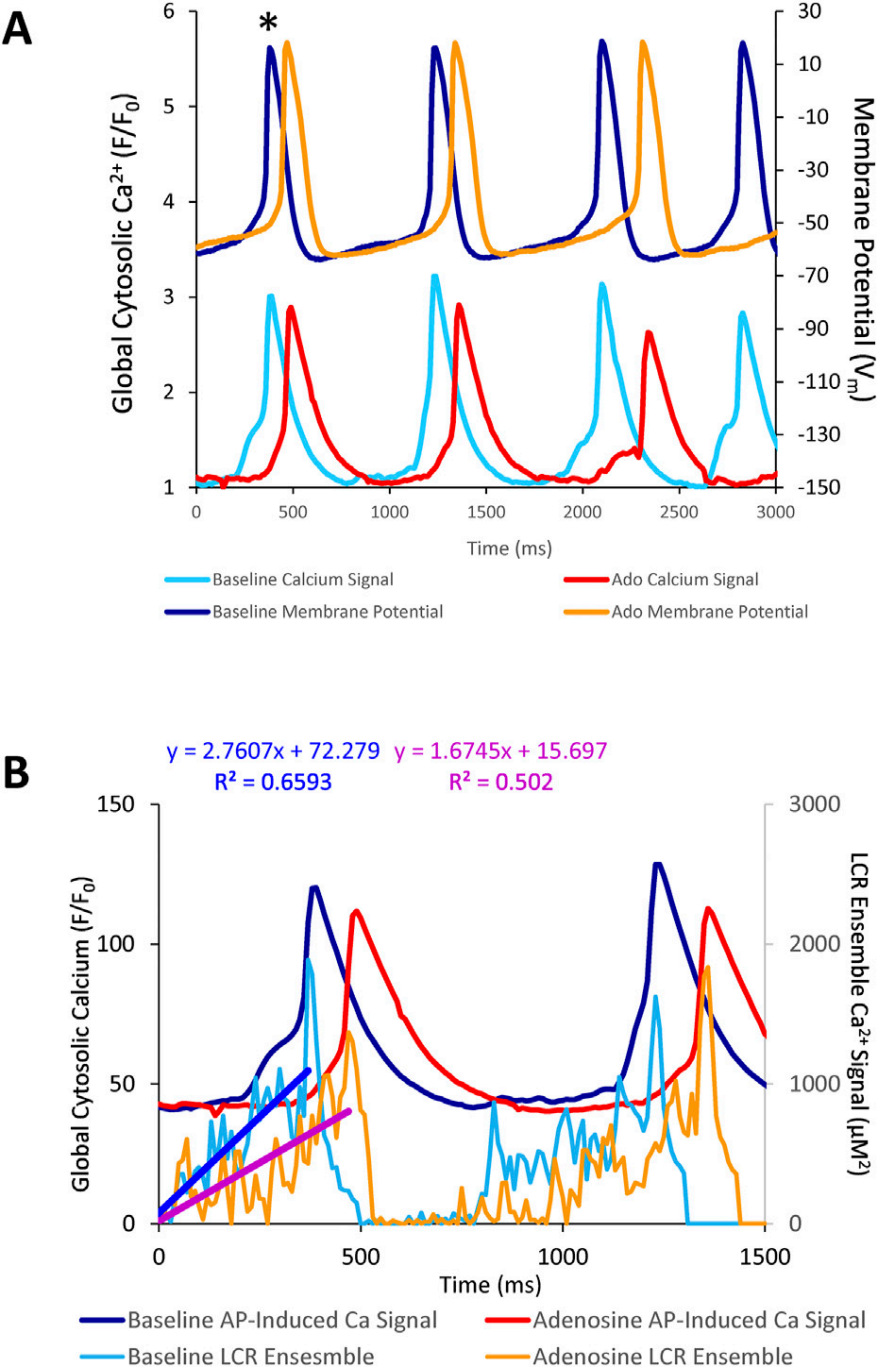
**(A)** simultaneous V_m_ and Ca^2+^ recordings of a rhythmically firing SANC at baseline that fires with decreased rhythmicity in response to 10 µM Ado. **(B)** the rate of LCR ensemble growth is steeper at baseline (blue line) and decreases in response to adenosine (magenta line). The asterisk indicates the baseline and ado cycle lengths measured in Figure 8A.

**FIGURE 9:**
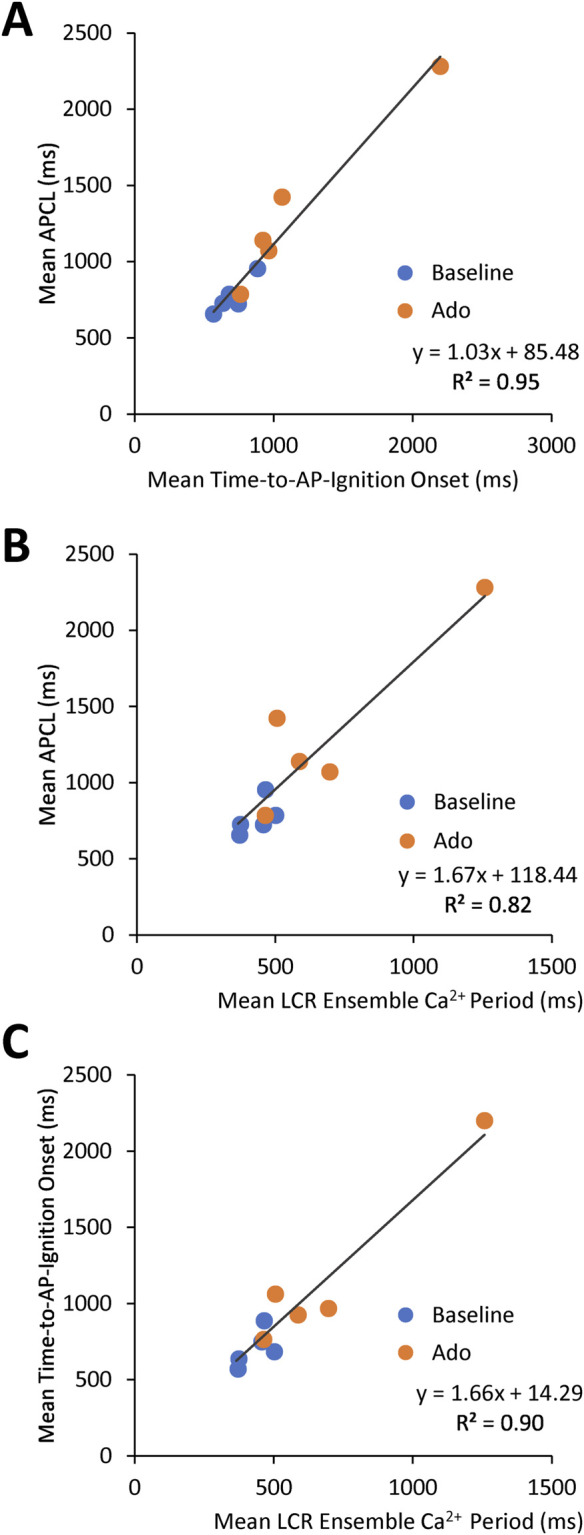
Relationships between key parameters of the coupled-clock system in control (baseline) and in the presence of adenosine in SANC in which Ca^2+^ and V_m_ were measured simultaneously. The LCR period informs on the APCL because the LCR period informs on the time-to-AP-ignition onset. Both Time-to-AP-ignition onset **(A)** and LCR period **(B,C)** and increase with adenosine.

The aforementioned measurements of Ca^2+^ and V_m_ were made in different cells. To directly assess the effect of ado on clock coupling, simultaneous membrane and Ca^2+^ measurements were performed within the same cell prior to and following ado superfusion (Supplementary Vedio S1; Figure 8). The prolongation and increased variability of APCL in the presence of ado occurred concurrently with a reduced growth rate of the LCR ensemble Ca^2+^ signal (Figure 9B). The AP ignition times, LCR periods, and APCLs of 5 cells in which V_m_ and Ca^2+^ were measured simultaneously are illustrated in Figure 9. The relationship between TTI and APCL in these 5 cells is illustrated in Figure 9A. Note how TTI and APCL increase in the presence of ado and maintain their relationship. TTI informs on the LCR period, even for extremely uncoupled cells (*r* ^2^ = 0.9) (Figure 9C) because the growth of the ensemble LCR Ca^2+^ signal, *via* its effect to increase more inward I_NCX_, initiates the ignition phase recorded as TTI (Lyashkov et al., 2018). Note a concurrent shift in TTI and LCR period at baseline and in the presence of ado (Figure 9C). Therefore, the APCL depends on the LCR period (Figure 9B) and that relationship informs on the fidelity of clock coupling: with ado, the clock coupling, reflected in the LCR period, was reduced.

### 3.7 V_m_-Ca^2+^ phase-plane diagrams

**FIGURE 10:**
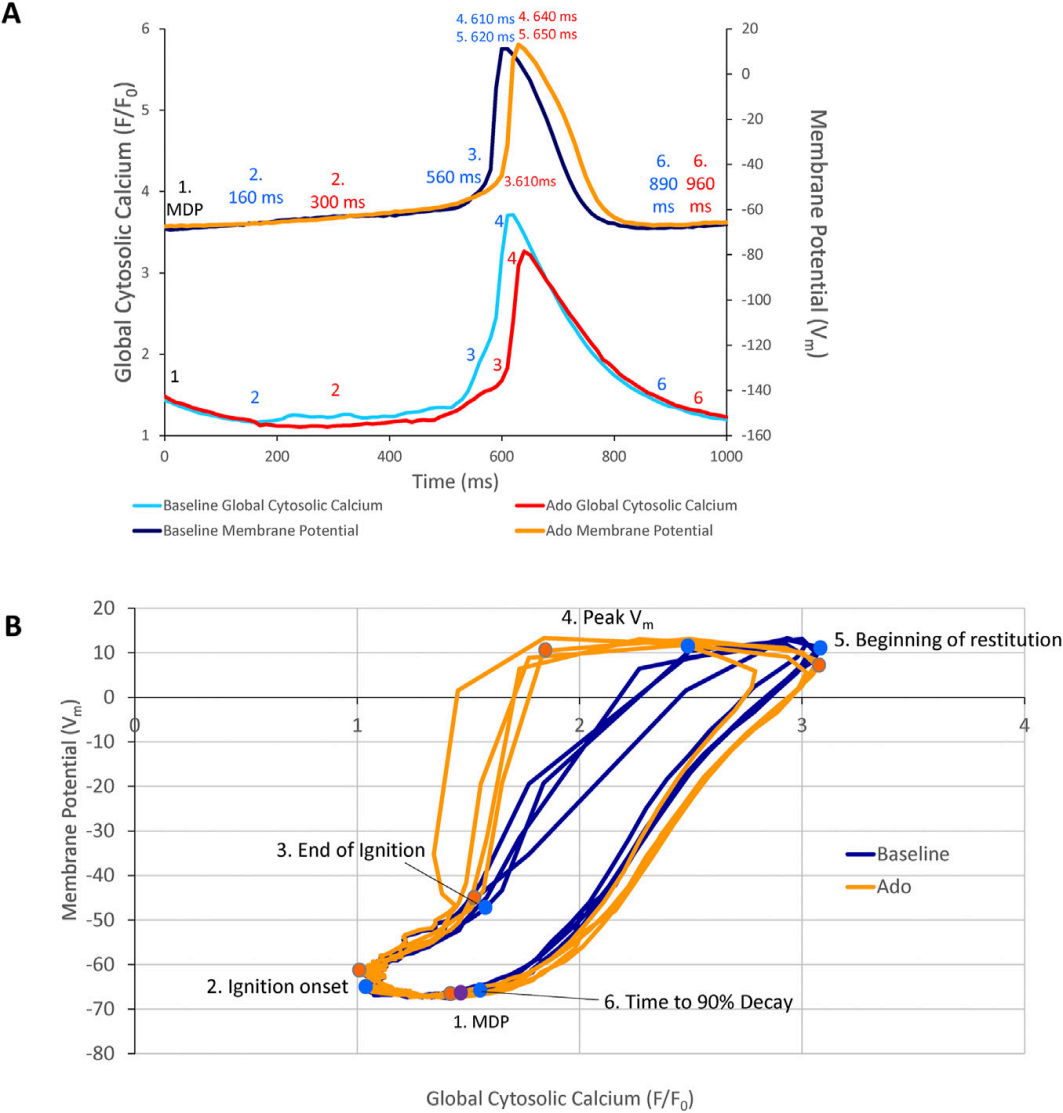
**(A)** Phase V_m_-Ca^2+^ relationship. **(A)** one AP cycle (* in Figure 5) prior to (baseline) and during adenosine (Ado) superfusion in which V_m_ and Ca^2+^ were measured simultaneously. **(B)** phase V_m_-Ca^2+^ diagram depicting the relationship between V_m_ and global cytosolic Ca^2+^ during several AP cycles (electrochemical gradient oscillations) at baseline (blue) and with adenosine (orange); 4 consecutive cycles from Figure 5 are shown. Numbers 1–5 indicate the times during the AP cycles in Panel **(A)**.

Phase-plane diagrams of V_m_ *versus* Ca^2+^ permit closer inspection of simultaneous time-dependent changes occurred in V_m_ and Ca^2+^. V_m_ and Ca^2+^ throughout the AP cycle inform on the electrochemical gradient oscillation that underlies each AP cycle (Lakatta et al., 2003). The phase-plane diagrams of the AP and Ca^2+^ recordings prior to and during ado superfusion are illustrated in Figure 10. Effects of ado on both clock parameters (Figures 3, 5; Tables 2, 3) would be expected to alter the electrochemical gradient oscillation characteristics exemplified by the V_m_-Ca^2+^ diagram.

Point 1 in Figures 10A,B marks the MDP in control and ado. The time from the MDP to ignition onset (labeled 2 in Panels A and B) occurred at a similar V_m_ and lower global Ca^2+^ in the presence of ado than at baseline. The AP ignition phase onset occurred earlier at baseline (160 msec) than in the same cells in the presence of ado (300 msec) (Figure 10A). Progressive Ca^2+^ ensemble self-organization and its effect on V_m_ and on the global Ca^2+^ signal caused the ignition process to proceed from 2 to 3, the take off potential of the AP, in Panels A and B. The take off potential marks the end of the ignition phase (labeled 3 in Panels A and B). The time from ignition onset to TOP (take off potential) is the duration of the ignition phase. Ado prolonged the time-to-ignition (MDP to ignition phase onset) from 160 msec at baseline to 300 msec with ado. The ignition phase duration decreased with ado from 400 msec at baseline to 310 msec with ado Pane A). Peak V_m_ and Ca^2+^ transient amplitudes (labeled 4 in Panels A and B) occur at later times with ado (640 ms) than baseline (610 ms) (Panel A). AP repolarization and Ca^2+^ transient decay initiates sooner at baseline than during ado (Panel A). Peak V_m_ amplitude, labeled 4 in Panels A and B, occurred at a lower Ca^2+^ level but later in time in ado (640 msec) than baseline (610 msec) (Figures 10A,B). The time to 90% restitution were 890 msec at baseline and 960 msec with ado at Point 6 (Panel A and B).

Note that the degree of hysteresis indicates the overall uncoupling of Ca^2+^ and V_m_ signals during an AP cycle. This hysteresis is greater with ado than at baseline. Whereas the difference of the duration of the AP ignition period is 560–610 msec (1.1 times greater) and time to 90% decay is 890 msec and 960 msec (1.08 times greater), the main difference to the time domain and percent differences between ado and baseline is the time-to-ignition onset. The major factor increasing hysteresis between ado and control in the phase-plane diagram is due to a 2-fold increase in the time-to-ignition onset in ado (300 ms) and control (160 ms). The time-to-ignition onset is regulated by the pumping of Ca^2+^ into the SR. This rate of Ca^2+^ cycling into the SR to achieve the threshold required for spontaneous LCRs to occur and self-organize, is the degree to which it influences V_m_ time-to-ignition onset.

### 3.8 Numerical modeling

Because the effects of ado were reported to be identical of those of ACh, we provide here (Figure 12, Supplementary Figure S1) results of our previous numerical model simulations that investigated the contributions of different mechanisms by which of ACh reduced SANC AP firing rate (Maltsev and Lakatta, 2010). Specifically, our numerical model included combined and separate contributions of I_CaL_, I_f_, I_KACh,Ado_ (membrane clock components) and SR Ca^2+^ pumping (Ca^2+^ clock), and demonstrated the their *synergistic effects* to reduce the AP firring rate (Figure 12). Our simulations also demonstrated the dynamic interplay of all mechanisms *via* coupled-clock mechanism in numerical model simulations during different AP phases (Supplementary Figure S1). It is important to note that the levels of both network SR (Ca_nSR_) and junctional SR (Ca_jSR_) substantially drop (bottom two panels in Supplementary Figure S1), explaining, in part, the decrease in Ca^2+^ transient amplitude in our experiments (Table 3 and Figure 5A).

## 4 Discussion

**FIGURE 11:**
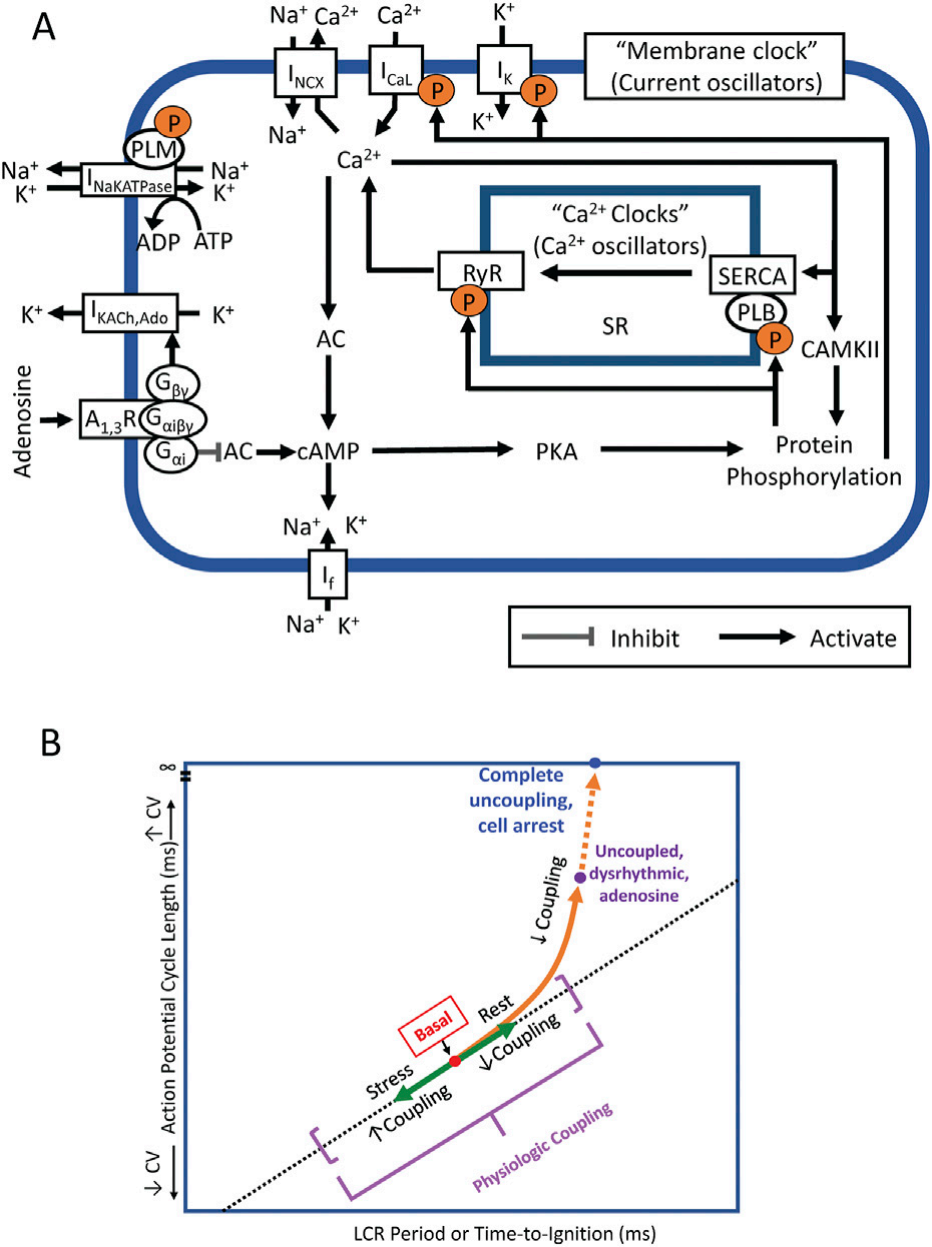
**(A)** Simplified schematic of the coupled system of membrane ion current oscillators and Ca^2+^ oscillators (Coupled-clock system) operative within sinoatrial nodal cells (SANC). The system provides robust and flexible AP firing rates. Constitutively active Ca^2+^-AC-cAMP-PKA signaling intrinsic to SANC, that is activated by Ca^2+^ oscillations, couples to an ensemble of electrogenic surface membrane molecules (current oscillators). AC, adenylyl cyclase; cAMP, cyclic AMP; PKA, protein kinase A; A_1_R, adenosine A_1_ receptor; G_αi,_ Gi protein alpha subunit; G_βγ,_ G_i_ protein beta gamma subunit; RyR, ryanodine receptor; SERCA, sarco-endoplasmic reticulum Ca^2+^-ATPase; PLB, phospholamban; SR, Sarcoplasmic reticulum; CaMKII, Ca^2+^/calmodulin-dependent protein kinase II; I_K_, delayed rectifier K^+^ current; I_KACh,Ado_, Acetylcholine/Adenosine-activated K^+^ current; I_f_, HCN currents; I_CaL_, L-type Ca^2+^ current; I_NCX_, Na^+^/Ca^2+^ exchanger currents; and I_NaKATPase_, Na^+^/K^+^ pump. **(B)** A schematic for SANC clock coupling/uncoupling. Clocks are coupled in the awake basal state, in response to physiologic stress (beta-adrenergic stimulation) coupling increases and beat-to-beat variability is reduced. In physiologic responses to vagal stimulation or adenosine, the clocks become partially uncoupled and the APCL becomes prolonged. These effects become more exaggerated as G_i_ coupled stimulation increases at higher drug concentrations.

The present study builds upon the known effects of ado to slow SANC AP firing and change V_m_ characteristics (Belardinelli et al., 1988). However, more recent studies have demonstrated that it is a coupled membrane and Ca^2+^ clock system that regulates SANC AP firing rate and rhythm. Ca^2+^ is an important oscillatory substrate involved in crosstalk between surface membrane and Ca^2+^ clocks (Figure 11A). The availability of intracellular Ca^2+^ is regulated by the balance of Ca^2+^ influx and efflux from the cell, which decrease in response to decreased clock protein phosphorylation. The present study is the first to measure and demonstrate that ado reduces and slows down intracellular Ca^2+^ cycling observed as a reduction in mean LCR size and duration and increased mean LCR period. The fidelity of clock coupling stems from the tight relationship between Ca^2+^ cycling and V_m_. Thus, ado regulates APCL and APCL variability by modulating Ca^2+^ and membrane clocks and their coupling (Figure 11B): the clocks become partially uncoupled and the APCL becomes prolonged (“physiological coupling”); these effects become more exaggerated as G_i_ coupled stimulation increases at higher drug concentrations, resulting in failed ignitions, arrhythmic AP firing, culminating in cell arrest upon complete uncoupling.

The present study demonstrates that ado increases APCL and APCL variability of SANC by directly and indirectly effecting both membrane and Ca^2+^ clocks. Ado directly activates I_KACh,Ado_ to hyperpolarize SANC and slow firing rate, thus directly effecting the membrane clock. Ado also has indirect effects on the membrane clock *via* G_αi_ (Figure 11A) to reduce intracellular protein phosphorylation and SR Ca^2+^ cycling to further slow SANC firing rate. Ado exerts direct effects on the Ca^2+^ clock by decreasing intracellular phosphorylation of Ca^2+^signaling proteins (like ACh). Ado indirectly effects the Ca^2+^ clock *via* its effect on the membrane clock to slow SANC firing rate, commensurate a decrease in net SR Ca^2+^ influx and intracellular Ca^2+^, i.e. oscillatory substrate for Ca^2+^clock. Thus, changes in SANC firing rate were due to direct effects on both clocks and indirect effects of both clocks on each other. Numerous feedbacks and feedforwards of this process occurred until a new equilibrium was reached.

Mechanisms by which ado slows AP firing and increases beat-to-beat variability are similar to cholinergic signaling *via* ACh. ACh and ado have different membrane receptors, but likely target the same I_KAch,Ado_ channels *via* G_βγ,_ hyperpolarizing the cell membrane and extending the time of diastolic depolarization (Kurachi et al., 1986). ACh, like ado, activates G_αi_ to inhibit AC activity and reduces cAMP-mediated, PKA dependent phosphorylation of downstream Ca^2+^ cycling proteins targets (DiFrancesco and Tromba, 1988; Dessauer et al., 1996; Lyashkov et al., 2009). While the present study did not directly measure ado’s effects on phosphorylation of clock proteins, it has been previously demonstrated that cholinergic receptor stimulation, like ado, decreases intracellular cAMP and phosphorylation *via* inhibition of AC activity (Lyashkov et al., 2009). Since ado acts *via* the same signaling pathway, its effects on intracellular Ca^2+^ cycling observed in this study likely result from the same changes to intracellular phosphorylation.

Other studies have demonstrated that because the membrane and Ca^2+^ clocks are tightly coupled, specific inhibition of a specific molecule in one clock that reduces SANC AP firing rate indirectly affects the function of the other clock and affects clock coupling fidelity. For example, specific I_f_ inhibition by ivabradine not only reduces SANC firing rate and increases AP cycle variability (Yaniv et al., 2014b), but also indirectly reduces intracellular Ca^2+^ (Yaniv et al., 2013). Thus, the overall Ca^2+^ effect of ivabradine, and likely ado, to reduce AP firing rate involves effects on both clocks, as well as clock coupling.

Cyclopiazonic acid (CPA) is a specific Ca^2+^ clock inhibitor that selectively and reversibly inhibits SERCA Ca^2+^-ATPase (Goeger et al., 1988; Nelson et al., 1994). CPA has been shown to dose-dependently decrease SANC firing rate by suppressing SERCA-mediated Ca^2+^ pumping (Vinogradova et al., 2010). The slowing of SANC firing with CPA was reflected by decreased SANC LCR size and number and increased LCR period (Vinogradova et al., 2010). The CPA-induced changes in LCR characteristics delayed the occurrence of LCR-activated I_NCX_ and reduced its amplitude, contributing to a decreased mean diastolic depolarization slope (Vinogradova et al., 2010). Thus, while CPA directly modified only Ca^2+^ clock function *via* the suppression of SERCA function, the collective effect on M and Ca^2+^ clock characteristics resulted in the APCL prolongation of the SANC firing. These results can be interpreted to indicate that any disturbances of the Ca^2+^ clock by ado in our experiments would also influence both clocks and their coupling, ultimately reducing SANC firing rate.

**FIGURE 12:**
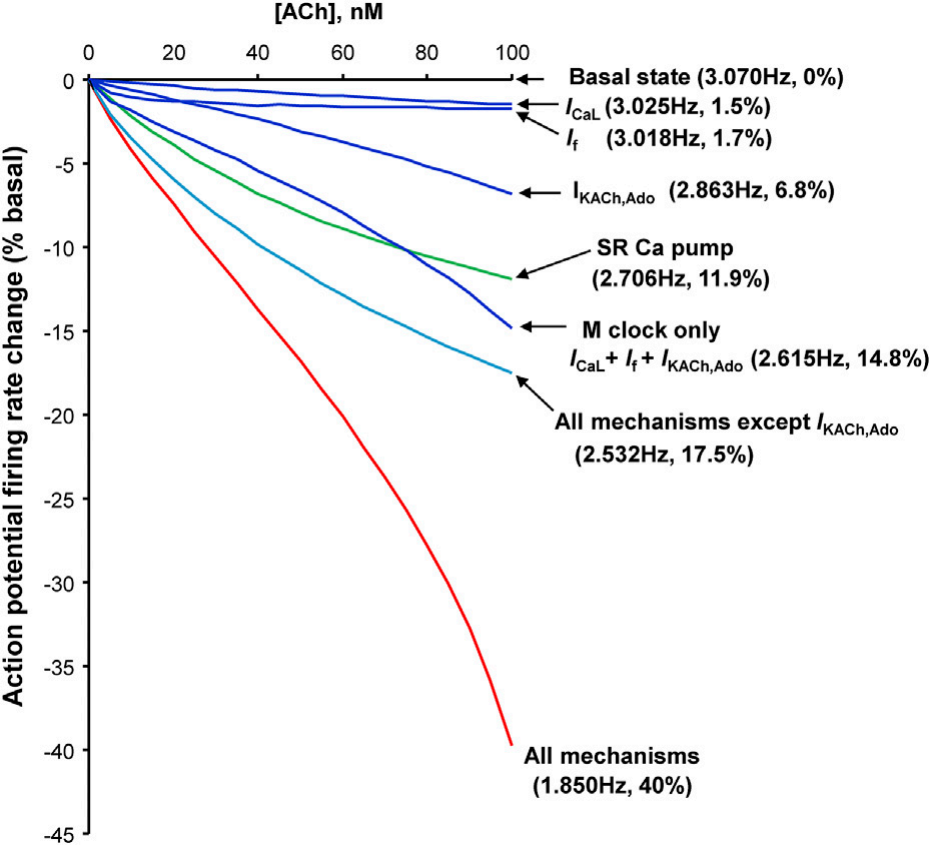
Synergism of different component mechanisms in SANC rate slowing by ACh. Results of numerical model predictions for spontaneous AP rate reduction at various moderate ACh concentrations (up to 100 nM) for different mechanisms (labels). Since ACh and ado act *via* the same signaling mechanism, a similar result is expected for ado. Modified from (Maltsev and Lakatta, 2010).

Our numerical modeling results (Figure 12, Supplementary Figure S1) provide insights into complex interplay of different downstream signaling mechanisms which are the same for ACh receptors and ado receptors (A_1_ and A_3_) coupled to G_i_ proteins (Figure 11A). Importantly, the effects of membrane clock and Ca^2+^ clock are synergistic (Figure 12). The effect of combined action of I_CaL_, I_f_ and I_KACh,Ado_ to decrease AP firing rate is 14.8%; the effect of SR Ca pump inhibition is 11.9%; however, the combined effect of all mechanisms is 40%. Our simulations also show that while I_NCX_ may not be directly affected by ACh or ado, it serves as a crucial functional link between the clocks in the rate slowing effects. Indeed, while in basal state firing the increase in Ca^2+^ release flux translates into respective increase of I_NCX_ (black double head arrow in Supplementary Figure S1), during stimulation the diastolic increase in the flux becomes missing and I_NCX_ also stays flat (red double head arrow), reflecting clock uncoupling. One important interplay happens with respect to SR Ca^2+^ loading. On the one hand, the longer SR Ca^2+^pumping *via* SERCA during the longer diastolic depolarization in the presence of ado stimulation favors a larger SR Ca^2+^ loading. On the one hand, ado *via* G_αi_ signaling attenuates AC activity that leads to lower levels of cAMP and cAMP-dependent PKA activity. This results in phospholamban de-phosphorylation that ultimately slows SERCA Ca^2+^ pumping (Figure 11A). Thus, while the SERCA pumping is longer during longer diastolic depolarization, it occurs at a slower rate. Furthermore, less frequent APs in the presence of ado provide, on average, less Ca^2+^ influx due to less frequent I_CaL_ activations. Also, during longer diastolic depolarizations forward mode NCX generates longer Ca^2+^ efflux. These factors (I_CaL_ and NCX) deplete cell of Ca^2+^. Thus, the factor of longer SERCA pumping competes with slower SERCA pumping and cell Ca^2+^ depletion. Our numerical modeling takes into account all these positive and negative contributions to SR Ca^2+^ loading and shows that SR Ca^2+^ content is expected to decrease, both the junctional SR and network SR (Supplementary Figure S1, two lower panels). Experimentally, the lower SR Ca^2+^ loading is reflected in a smaller, on average, AP-induced Ca^2+^ transient peak amplitude (Table 3, column “Peak Ca^2+^“).

Our numerical modeling simulations (Figure 12) are in line with previous experimental studies of ACh effects (Lyashkov et al., 2009): At low (more physiological) ACh doses, we observed that a specific blockade of I_KACh_,_Ado_ by tertiapin had no effect; its effect begins to occur at higher concentration of ACh, i.e., at about the half maximum dose of ACh (Supplementary Figure S2). Thus, these results can be interpreted to indicate that inhibition of the Ca^2+^ clock in isolated single SAN cells seems to contribute substantially at physiological (low) activation of muscarinic cholinergic receptor (or adenosine receptors), at which I_KACh,Ado_ is not activated. It is important to note, however, that interpretation of our experimental and numerical modeling results obtained in single SANC to SAN tissue is complicated. For example, it was shown (Li et al., 2017) that specific blockade of I_KACh,Ado_ by tertiapin substantially reduces the effect of subsequent application of ado in SAN of some individual human hearts, pointing to a possible substantial contribution of I_KACh,Ado_ mechanism in ado effect in SAN cells. However, a problem with such interpretation, specifically attempts to find a leading mechanism of ado effect, is that SAN is extremely heterogeneous tissue (Boyett et al., 2000; Bychkov et al., 2022). Autonomic ganglia embedded in the SAN epicardium connect the nerves that densely innervate SAN tissue, releasing neurotransmitters biding to autonomic receptors on the cells that modulates the beating rate of these cells (Vincenzi and West, 1963; Jalife et al., 1983). Furthermore, SANC generate heterogeneous signaling within SAN tissue (Bychkov et al., 2020), driven by the coupled-clock system in each cell with numerous interacting components of both clocks (Lakatta et al., 2010). An inhibition of one clock inevitably perturbs the other clock (Yaniv et al., 2013). How heterogeneous properties of individual cells within SAN tissue give rise to synchronized, rhythmic APs at the SAN exits remains a mystery and represents the frontier of the cardiac pacemaker research (Mata et al., 2019; Bychkov et al., 2020; Clancy and Santana, 2020; Fenske et al., 2020; Weiss and Qu, 2020; Bychkov et al., 2022; Campana et al., 2022; Guarina et al., 2022; Maltsev et al., 2022).

Adenosine is known to be released in response to metabolic stress such as hypoxia and inflammation (Grenz et al., 2011; Idzko et al., 2014). Upregulation of A_1_R protein expression and increased plasma levels of ado are also found to be associated with heart failure and ischemia (Newman et al., 1984; Funaya et al., 1997; Lou et al., 2014). Evidence is accumulating that adenosine contributes to SAN dysfunction in heart failure (Lou et al., 2014). New insights, through 2D optical mapping of the intact SAN, have shown that failing SAN is increasingly sensitive to ado (Lou et al., 2014; Li et al., 2017). This increased influence of ado has been found to contribute to SAN dysfunction by amplifying intrinsic conduction abnormalities such as atrial fibrillation and sinus exit block (Li et al., 2017). Given the significant contribution of adenosine to SAN dysfunction, its effect on isolated SANC *in vitro* shown here may have some baring on arrhythmias in the context of heart failure and ischemia.

We performed experiments in single SANC isolated from rabbits that is a classical species to investigate pacemaker mechanisms. On the other hand, cAMP-mediated effects of ado have been strongly associated with a level of sympathetic stimulation that is different in different species at baseline. It is also important to note that small rodents (mainly used for genetic manipulations) have a high surface-to-body mass, causing them to lose a large amount of heat. In order to maintain 37°C mice and rats employ nonshivering thermogenesis, which activates sympathetic stimulation to burn brown fat to produce heat. A biproduct of the excessive sympathetic dischargers is the elevation of heart rate in small rodents (mice and rats). For example, maintaining mice in thermo-neutral metabolic environment 30°C vs room temperature 20–24°C markedly reduces the heart rate as much as 40% (Axsom et al., 2020).

## 5 Study limitations and future studies

The AP-induced Ca^2+^ transient cycle length was, on average, 13% longer in cells loaded with Ca^2+^ indicator Fluo-4 than cells not loaded with Fluo-4. This may reflect Ca^2+^ indicator buffering intracellular Ca^2+^. Nevertheless, ado effects on mean AP-induced Ca^2+^ transient and APCL variability were similar to the effects of ado on APCL and APCL variability recorded *via* perforated patch clamp in the absence of Ca^2+^ indicator.

While the aim of the study was to address the coupled-clock system, specifically LCRs and the membrane potential in ado effects, the biochemical pathways and interplay of LCRs with I_K,Ado_, I_CaL_, I_f_ and other specific mechanisms and molecules have not been experimentally studied here and merit future investigations. As mentioned above, cAMP-mediated effects of ado have been strongly associated with a level of sympathetic stimulation. Therefore, Ca^2+^ signaling (Ca^2+^ clock) could be also important in the setting of sympathetic stimulation and represents an important topic for future studies.

One important SAN phenomenon is the shift of pacemaker location within the SAN tissue in response to different perturbations, including ado (Li et al., 2017). While our single cell data cannot be directly interpreted to explain this and other phenomena on the tissue level, the intrinsic properties of SAN cells can be used in future numerical model simulations (Mata et al., 2019; Campana et al., 2022; Maltsev et al., 2022) to get insights into SAN tissue function and ado effects. Importance of Ca^2+^ signaling for tissue function, and especially for the shift of pacemaker location, has been demonstrated in recent studies of S100B effects in intact SAN (Bychkov et al., 2022).

Finally, it is important to note that while we traditionally discuss average data and illustrate the mechanisms in a numerical model of a SANC, in reality, SANC are a naturally heterogenous population of cells (Musa et al., 2002; Monfredi et al., 2018) that have different combinations and contributions of specific mechanisms within the cell population for autonomic modulation [see for example, (Kim et al., 2021; Louradour et al., 2022)]. Areas within SA node that are insensitive to ACh (and likely to ado) have been reported by Opthof (Opthof, 2007). This heterogeneity is reflected in our data by different degree of MDP hyperpolarization and cycle length increase in response to ado. Characterization of the population response, rather than an “averaged cell” response is crucially important for future studies, not only of ado mechanisms but of any intervention of SANC function. This approach becomes even more imperative and promising in the light of recent paradigm shift of SA node function with respect to heterogeneous local signaling, including different cells populations with no AP firing and subthreshold signaling (Bychkov et al., 2020; Clancy and Santana, 2020; Fenske et al., 2020; Weiss and Qu, 2020; Guarina et al., 2022). Thus, based on the new data and approaches, our study has a limitation that we measured only rhythmically beating cells and our measured cell numbers in different sets of experiments varied from 4 to 46 that is insufficient to characterize responses of cell populations. Future studies of responses in a larger cell population of isolated cells will help to better understand the complex cellular mechanism of ado action.

## Data Availability

The raw data supporting the conclusion of this article will be made available by the authors, without undue reservation.
