# Supplementary material for: Adenosine reduces sinoatrial node cell action potential firing rate by uncoupling its membrane and calcium clocks
Source: Front Physiol. 2022 Nov 24;13:977807. doi: 10.3389/fphys.2022.977807 (PMC9730041; doi:10.3389/fphys.2022.977807)
Supplement: Supplementary file 1 [file DataSheet2.PDF]

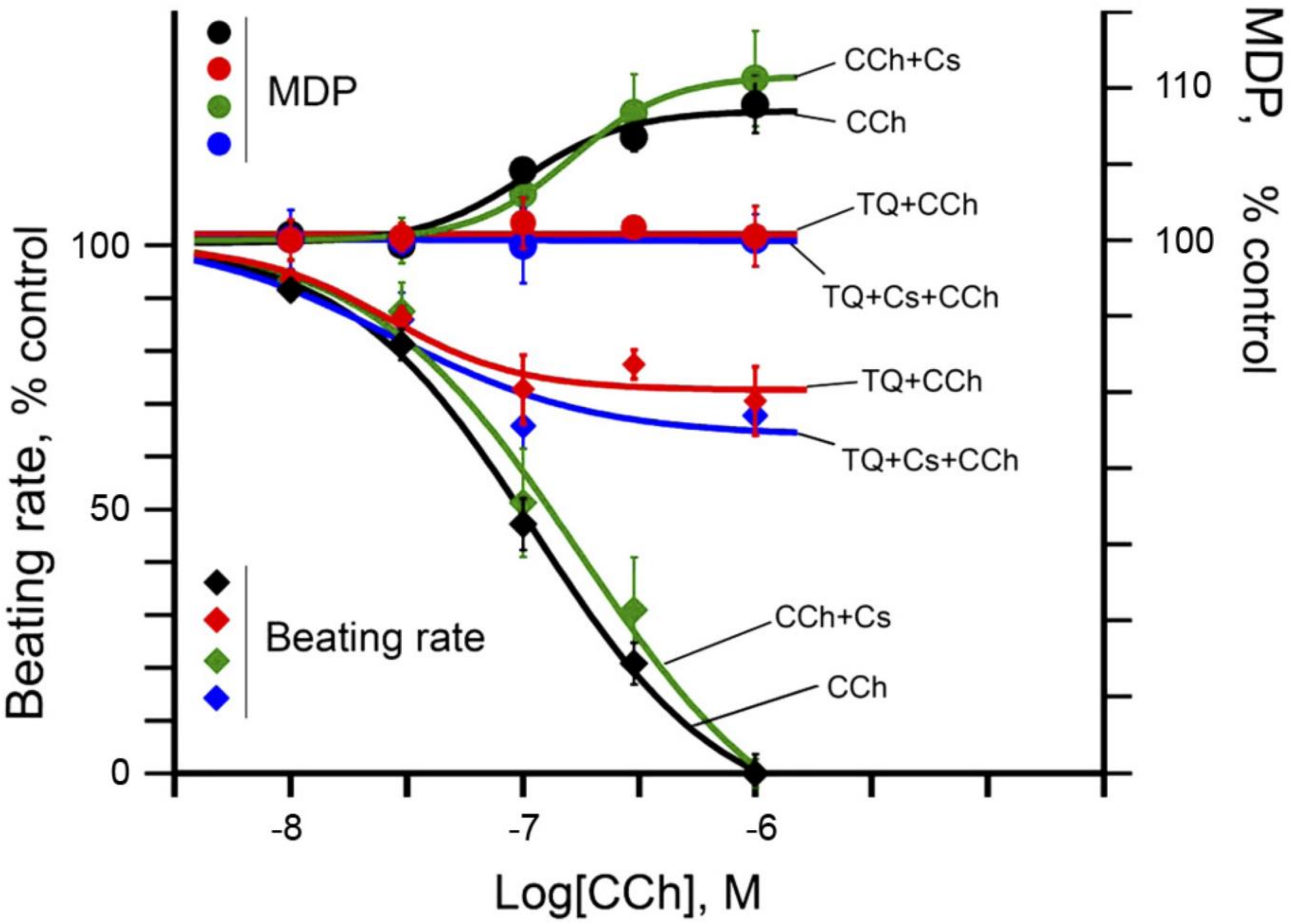


**Figure S2.** Average dose-dependent peak effects of carbachol (CCh) on maximum diastolic potential (MDP) and beating rate reduction (BRR) in spontaneously beating sinoatrial nodal cells (SANC) and in SANC with blocked $I_f$ by CsCl or blocked $I_{KACh,Ado}$ by tertiapin Q (TQ) or block of both currents simultaneously (CCh, n =43; TQ+CCh, n= 22; CsCl+CCh, n=22; TQ+CsCl+CCh, n=12). Each cell received only a single dose of CCh. Since ACh and ado act via the same signaling mechanism, a similar result is expected for ado. Modified from Lyshkov et al. 2010. *American Journal of Physiology* 2009; 297:H949-H959.
