# Supplementary material for: Adenosine reduces sinoatrial node cell action potential firing rate by uncoupling its membrane and calcium clocks
Source: Front Physiol. 2022 Nov 24;13:977807. doi: 10.3389/fphys.2022.977807 (PMC9730041; doi:10.3389/fphys.2022.977807)
Supplement: Supplementary file 2 [file DataSheet1.PDF]

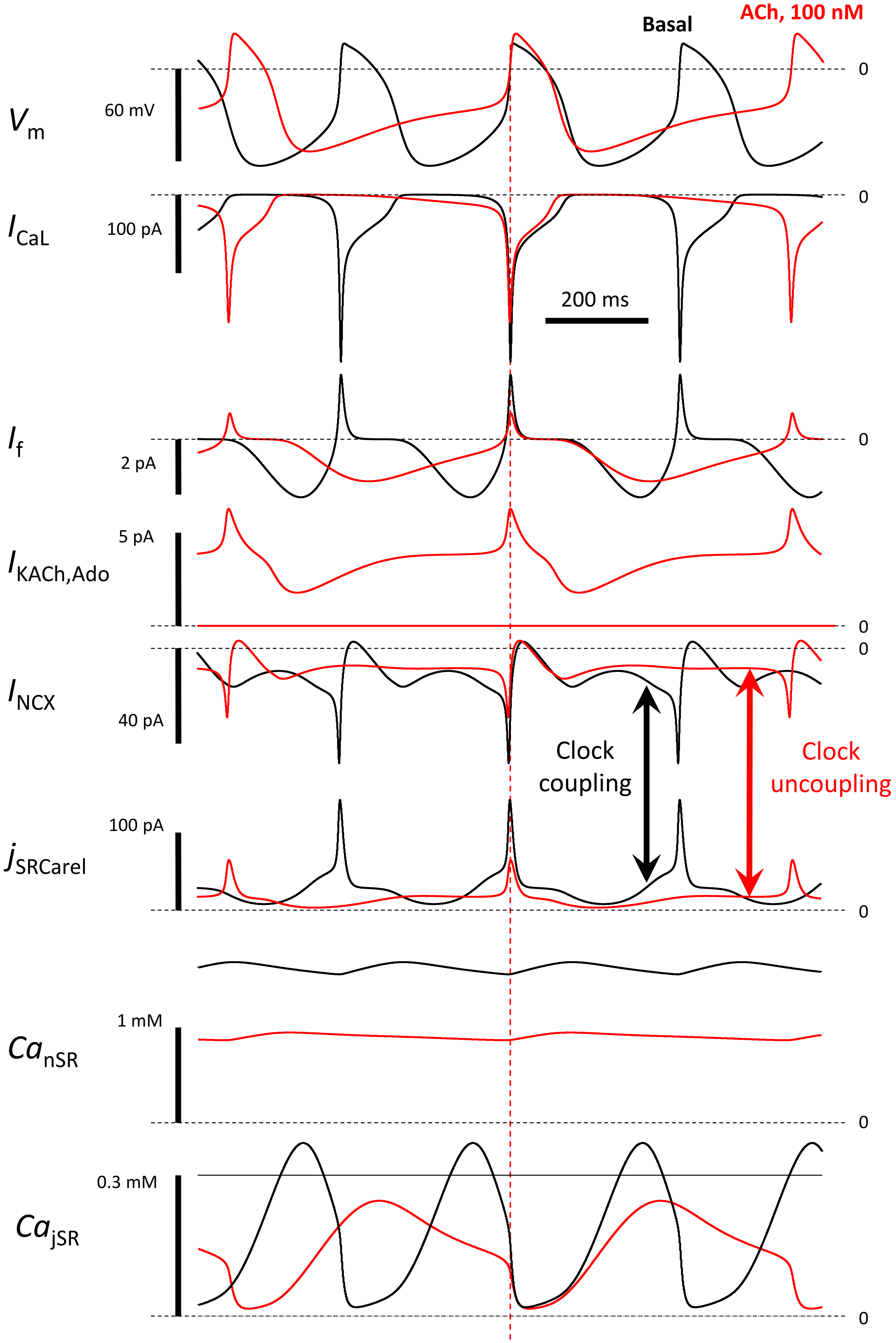

Figure S1. Model simulations illustrating the mechanism of ChR stimulation effect that integrates function of components of Ca^{2+} clock and membrane clock. A major mechanism of cycle length increase, in addition to activation of $I_{KACh,Ado}$, is a later and smaller I_{NCX}, i.e. less clock coupling (red arrow vs. black arrow). Also, the smaller and less frequent I_{CaL} activation affects the AP firing by supplying less Ca^{2+} to the cell and thereby decelerating SR function. The traces are synchronized at the phase of V_{m}=0 (vertical dash line). j_{SRCarel} is the Ca^{2+} release flux that reflects the LCR ensemble signal in diastolic phase and Ca transient is systolic phase. Since ACh and ado act via the same signaling mechanism, a similar result is expected for ado. Modified from Maltsev and Lakatta. 2010. *American Journal of Physiology*. 298:H2010-H2023. Data in panel for network SR loading with Ca^{2+} (Ca_{nSR}) has not been previously published.
